# Innovative Target for Production of Technetium-99m by Biomedical Cyclotron

**DOI:** 10.3390/molecules24010025

**Published:** 2018-12-21

**Authors:** Hanna Skliarova, Sara Cisternino, Gianfranco Cicoria, Mario Marengo, Vincenzo Palmieri

**Affiliations:** 1 Legnaro National Laboratories, Italian National Institute for Nuclear Physics (LNL-INFN), Viale dell’Università 2, 35020 Legnaro PD, Italy; Sara.Cisternino@lnl.infn.it; 2 Medical Physics Department, University Hospital “S. Orsola-Malpighi”, 40100 Bologna, Italy; cicoria.gianfranco@aou.mo.it (G.C.); mario.marengo@unibo.it (M.M.)

**Keywords:** cyclotron target, radiopharmaceutical production technology, magnetron sputtering, vacuum brazing

## Abstract

Technetium-99m (^99m^Tc) is the most used radionuclide worldwide in nuclear medicine for diagnostic imaging procedures. ^99m^Tc is typically extracted from portable generators containing ^99^Mo, which is produced normally in nuclear reactors as a fission product of highly enriched Uranium material. Due to unexpected outages or planned and unplanned reactor shutdown, significant ^99m^Tc shortages appeared as a problem since 2008 The alternative cyclotron-based approach through the ^100^Mo(p,2n)^99m^Tc reaction is considered one of the most promising routes for direct ^99m^Tc production in order to mitigate potential ^99^Mo shortages. The design and manufacturing of appropriate cyclotron targets for the production of significant amounts of a radiopharmaceutical for medical use is a technological challenge. In this work, a novel solid target preparation method was developed, including sputter deposition of a dense, adherent, and non-oxidized Mo target material onto a complex backing plate. The latter included either chemically resistant sapphire or synthetic diamond brazed in vacuum conditions to copper. The target thermo-mechanical stability tests were performed under 15.6 MeV proton energy and different beam intensities, up to the maximum provided by the available GE Healthcare (Chicago, IL, USA) PET trace medical cyclotron. The targets resisted proton beam currents up to 60 µA (corresponding to a heat power density of about 1 kW/cm^2^) without damage or Mo deposited layer delamination. The chemical stability of the proposed backing materials was proven by gamma-spectroscopy analysis of the solution obtained after the standard dissolution procedure of irradiated targets in H_2_O_2_.

## 1. Introduction

Technetium-99m (^99m^Tc) is an extremely important radionuclide, used in the vast majority of traditional diagnostic SPECT (Single Photon Emission Computer Tomography) imaging examinations. The radioisotope is usually available in hospitals from portable generators containing the ^99^Mo parent nuclide, a fission product of highly ^235^U-enriched uranium. About 95% of the ^99^Mo world production is provided by a few ageing nuclear reactors, whose unplanned outages have already caused shortages at the global level, compromising the availability of ^99m^Tc. In order to compensate potential ^99^Mo shortages in the future, alternative accelerator-based routes for the production of ^99m^Tc have been intensively developed.

The theoretical and experimental results obtained at the global level [1,2,3,4,5,6] confirmed that the direct cyclotron-produced ^99m^Tc, through the ^100^Mo(p,2n) nuclear reaction route starting from highly ^100^Mo-enriched (i.e., >99% at.) molybdenum targets, is the most promising approach. It has been determined that the optimal proton energy range is 10 to 22 MeV since it is able to provide sufficient amounts of the radioisotope for hospital needs with impurity levels within the limits defined by the recently issued European Pharmacopeia [7]. The ^100^Mo target material has to be in the range of hundreds of microns [2]. Depending on the irradiation conditions, irradiation time, beam spot size, Mo enrichment level, and target thickness, the activity produced in a single run can vary and reach up to about 350 GBq [3,6,8,9].

For a number of different cyclotron target stations, various target systems have been developed and tested. The use of enriched and natural Mo under metallic, oxide, and carbide forms deposited on different backing has been considered [4,5]. Due to the poor thermal conductivity of molybdenum oxide, which limits the maximum amount of applied beam current, only metallic Mo targets should be considered for high power irradiation needed for large-scale ^99m^Tc production.

During the target design process, two critical issues have to be considered to meet the requirements for radiopharmaceutical production: (1) Beam power heat dissipation capacity has to be as high as possible to exploit the available cyclotron beam current in order to achieve maximum production yield and (2) the baseplate has to be inert during the following target dissolution to provide the requested chemical and radiochemical purity of the final product. The design and construction of a solid target prototype that satisfies both requirements is a challenge.

In order to guarantee an efficient dissipation of the heat imported by the beam, the Mo metallic target must have the following properties: good thermal conductivity of Mo and backing material, uniform and controlled thickness of Mo, and high density (bulk-like) and low level of oxidation of Mo.

The LARAMED (LAboratory of RAdioisotopes for MEDicine) group at LNL-INFN (Legnaro National Laboratories, Italian National Institute for Nuclear Physics) has proposed the use of magnetron sputtering (MS) as ^100^Mo deposition technique onto a backing plate as sputtering is able to produce both high-density target material and good adherence onto a backing plate. MS deposition allows good control of the deposited layer thickness.

MS is a physical vapor deposition (PVD) technique, well-known for deposition of thin metallic films. However, MS is not used for thick film deposition because of tensile or compressive stress always present in the films. In this work, a method to directly deposit dense and stress-free Mo metallic films up to hundreds of microns thick onto a backing plate was developed. This approach mitigates the often under-evaluated problem of the thermal contact between the target and target backing plate.

The most common approach for Mo target processing after irradiation is target dissolution in concentrated H_2_O_2_ at elevated temperatures (70–90°C), followed by the application of one of the already developed methods to separate ^99m^Tc from ^100^Mo and other radioisotopes [10]. Since concentrated hydrogen peroxide at elevated temperatures is a corrosive agent, a proper backing plate material should be chosen according to its chemical inertness in such conditions in order to minimize possible impurities in the final product.

For this reason, sapphire, a ceramic material with one of the highest thermal conductivities (60 Wm^−1^K^−1^), and synthetic diamond (CVD), a material with an extremely high thermal conductivity (up to 2000 Wm^−1^K^−1^), are proposed in the present work as the components of the target backing plate. In order to minimize the thermal resistance related to the ceramic part (again, to optimize the heat exchange of the target), its thickness was minimized. To simultaneously maintain the mechanical rigidity of the system and minimize costs, the ceramic substrates were brazed onto a metallic target holder part (copper). Only one side of the target prototype is exposed to the dissolution agent due to a dedicated bottom-opening dissolution vial.

Both critical issues faced by the cyclotron solid target have been resolved and are presented here as a proposal for innovative target preparation, which consists of the deposition of more than 100 µm-thick Mo film by magnetron sputtering onto a sapphire or synthetic diamond substrate, brazed to a high thermal conductivity metallic holder.

## 2. State-of-the-Art: Cyclotron Target for ^99m^Tc Production

Although research and development (R&D) activity on ^99m^Tc cyclotron production using oxide [4,5,11,12,13] carbide [4,5] and even aqueous solution [4,5] targets has been extensive, metallic Mo solid targets are considered the most promising, as they are able to provide the highest production yield. That is due to the high level of heat dissipation associated with the higher thermal conductivity of the metal. Even traces of oxygen can drastically reduce the thermal conductivity of the target material.

Two approaches—preliminary Mo pellet preparation followed by subsequent bonding to a backing plate and direct deposition of Mo onto a backing—have been used.

^100^Mo-enriched metallic molybdenum is usually available on the market in powder form. Thus, the transformation of refractory metal powders into dense pellet or foil has been studied by different research groups applying a range of different methods, including pressing, sintering, and rolling. The targets produced using only hydraulic pressing of powder [4,5,14,15,16] are characterized by much lower density than their corresponding bulk material and can sustain relatively limited cyclotron currents. The sintering of Mo powders [4,9,17] followed by press bonding [4,5,14,15,16] or vacuum brazing [4,9,17] processes were used to manufacture Mo targets, sustaining beam currents in the order of hundreds of µA.

Targets for nuclear cross-section measurements were produced by press-rolling, starting from powders or from Mo beads produced by e-beam powder melting [4,5,18], otherwise by re-melting of Mo powders, followed by press-roller reshaping. However, this method is not applicable to routine production in which a thick target is required, as the method is unable to provide a good thermal contact among Mo stacked foils or between them and the backing during irradiation. Instead, using direct Mo deposition onto a backing plate, the second step (i.e., pellet bonding to a backing plate) is unnecessary. The easiest approach (i.e., Mo powders melting directly onto a backing plate [19]), has been also investigated, but the results have shown non-uniformity of the Mo layer.

Electroplating of metals from aqueous solutions is a well-known industrial process. However, refractory metals such as Mo are difficult to deposit using standard electrodeposition methods due to their high affinity for oxygen. Despite this, electroplating from particular alkaline [20] or acetate [21] solutions has been described. Notably, such deposits have thicknesses not exceeding 20 µm and high levels of oxidation. In addition, the described process was found to be very inefficient (<2%). The co-deposition of molybdenum with zinc [22] was reported to be a much more efficient approach. The electrodeposition from ionic liquids or molten salts [4,23,24] has produced better Mo layer quality, although the use of expensive equipment and implementation of a more complicated protocol were required. Electrophoretic deposition from a mixture of Mo powders and molybdate with additives, followed by subsequent sintering at high temperature, has produced sufficient Mo thickness and resistance up to 300 µA [5,25]. Further improvement of the process by sintering in an inert atmosphere [4] has provided targets resistant up to 500 µA. Notwithstanding the much better results in beam power tests, the accumulation of impurities from the electrolyte bath is, however, an important drawback of all electrochemical deposition methods that is hard to overcome in target manufacturing.

The PVD methods for direct Mo deposition described in the literature include thermal spray [26], cathodic arc [4], argon [27] and xenon [28] focused ion beam (FIB) sputtering, but no information about cyclotron beam tests of such targets is available. Even if only less than 1-µm-thick Mo coatings by FIB sputtering have been reported, the method still seems interesting because it allows extremely low target material amounts, low losses of expensive isotope-enriched material during deposition, and high purity target material. The LARAMED group at LNL-INFN has been working on magnetron sputtering as a method for direct Mo deposition since 2013 [5].

A number of materials have been proposed and tested as a backing plate for the cyclotron ^99m^Tc production target. Among them, the most popular are copper [4,5,26,29], aluminum [4,5,14,15,16,18], tantalum [4,5,14,15,16,19,25], GLIDCOP (Höganäs AB, Höganäs, Sweden) [4,9,17], and platinum [4,5,20].

Considering the reference ^100^Mo(p, 2n)^99m^Tc production route, the standard target processing procedure includes dissolution of a Mo and Tc mixture in hot concentrated H_2_O_2_. Transition, post-transition, and refractory metals are not perfectly inert at such conditions. In the case of radiopharmaceutical production, even the presence of a very low amount of impurities is critical.

The idea to use an inert baseplate was presented by the group at the University of Alberta during the International Atomic Energy Agency (IAEA) meeting held in 2013 [5] and 2015 [4], devoted to the accelerator-based production of ^99m^Tc, including materials such as glassy carbon, quartz, and aluminum oxide. At the same meetings, a Japanese group proposed the use of an inert vessel (aluminum oxide [5], SiC [4]) for direct irradiation of Mo powders by a vertical cyclotron beam.

Table 1 presents a comparative summary of the studies carried out in different countries on targetry and target preparation for ^99m^Tc production by a cyclotron, highlighting the main drawbacks of each approach.

Even if progress is being accomplished on Mo solid target design and optimization all over the world, the problem of the target sustaining elevated cyclotron currents and providing few impurities is still a challenge. In this work, we propose solving this problem using Mo direct deposition by magnetron sputtering onto a complex backing plate composed of chemically inert sapphire or synthetic diamond (only this part is exposed during dissolution to H_2_O_2_ dissolution media), brazed to a high thermal conductivity metallic holder.

## 3. Materials and Methods

### 3.1. Materials

Commercial sputtering targets with naturally abundant Mo (99.99% purity; Mateck GmbH, Julich, Germany) and naturally abundant Ti (99.99% purity; Goodfellow Cambridge Ltd., Huntingdon, England) were used for films deposition. Argon at 99.998% purity (SIAD S.p.A., Bergamo, Italy) was used as the sputtering gas during the process.

Copper disks 32 mm in diameter and 1.5 mm thick were used as simple target backing and substrates for direct Mo sputtering. Copper components, which were disks 32 mm in diameter and 1.5 mm thick with a cylindrical recess 0.5 mm deep and diameter corresponding to the size of sapphire/synthetic diamond plus brazing radial clearance, were used for the preparation of complex target prototypes.

The standard procedure for copper substrate preparation included: ultrasonic washing for 20 min with GP 17.40 SUP soap (NGL Cleaning Technology SA, Nyon, Switzerland) and deionized water, chemical etching with SUBU5 solution (5 g/L sulfamic acid, 1 g/L ammonium citrate, 50 mL/L butanol, 50 mL/L H_2_O_2,_ and 1 L deionized water) at 72 ± 4 °C in order to remove surface oxides, passivation in 20 g/L sulfamic acid, ultrasonic washing with water for 20 min, rinsing with ethanol, and drying with nitrogen.

IR-grade sapphire with C-axis orientation (Meller Optics Inc., Providence, RI, USA) discs 12.7 mm in diameter and 0.5 mm thick, and chemical vapor-deposited (CVD) synthetic diamond substrates (II–VI Advanced materials GmbH, Pine Brook, NJ, USA) 13.5 mm in diameter and 0.3 mm thick of thermal grade, with thermal conductivity 1500 W/(m·K) were used for target backing preparation

For pre-treatment of all non-metallic substrates, including sapphire, synthetic diamond, and the silicon wafer, the same procedure was used: ultrasonic washing for 20 min with Rodaclean^®−^(NGL Cleaning Technology SA, Nyon, Switzerland) soap and then deionized water for 20 min, rinsing with ethanol, and drying with nitrogen flux.

Copper disks 13 mm in diameter and 1 mm thick were used as substrates for Mo sputtering for the Scanning Electron Microscopy (SEM) analysis of Mo film. For the SEM cross-section analysis, the samples were cut by electro-erosion, treated with abrasive paper, and then chemically etched in a “base piranha” solution (H_2_O + NaOH + H_2_O_2_ 1:1:1 vol.) for 1 min at 50 °C.

### 3.2. Cyclotron

**Figure 1:**
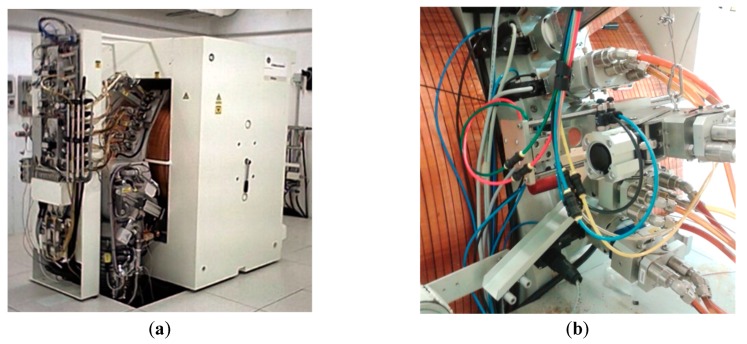
(**a**) GE PETtrace cyclotron at S. Orsola-Malpighi Hospital, Bologna; (**b**) solid target station at Sant’Orsola-Malpighi Hospital, Bologna.

In this study, a GE PETtrace 800S cyclotron (GE Healthcare, Chicago, IL, USA), installed at S. Orsola-Malpighi Hospital in Bologna, was used for all irradiation tests. The PETtrace (Figure 1a) is an isochronous cyclotron working at fixed energy (16.5 MeV for protons and 8.4 MeV for deuterons) and a maximum beam intensity of 100 μA. Note, the maximum current available depends on the source and tuning of the magnets.

The solid target station (prototype TEMA Sinergie S.P.A., Faenza, Ra, Italy) used in this work is shown in Figure 1b. The target “coin” is manually placed inside the target station before irradiation. The aluminum water cooling chamber is pressed to the back of the target coin using a ~3 bar air pneumatic piston. After irradiation, the system allows an automatic target transfer outside the cyclotron bunker. Double cooling is used to increase the current allowed on the target: water-cooling from the back, and helium gas cooling from the front side of the target coin. A detailed description of this irradiation unit was reported previously [32].

The target prototypes were developed fitting the design of this target station. The typical target coin is a disk at most 32 mm in diameter and 2 mm thick. In the current solid target system, the integer target coin provides maximum heat exchange performance that is limited by the thermal resistance of both the deposited Mo layer and the backing plate, and the contribution due to the thermal contact resistance between them.

**Figure 2:**
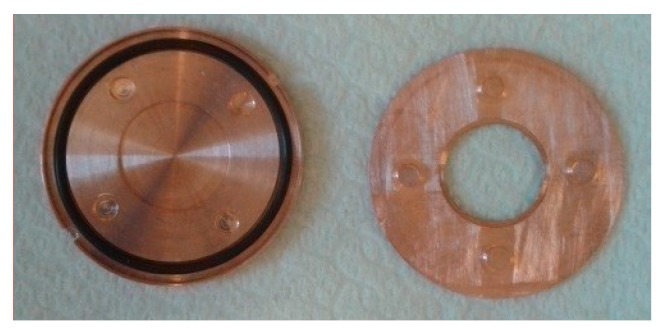
Four-pin clamping target system.

For this work, a specific 4-pin target holder was designed (Figure 2) to house the sapphire and synthetic diamond disks for testing chemical inertness. The external dimensions of the target holder were 32 mm diameter and 2 mm thick once assembled.

The target prototypes for thermomechanical stability control were irradiated for one minute (enough to reach thermal equilibrium) with increasing current. After each irradiation, the sample was unloaded and the integrity of the target and the adhesion of the Mo film on the backing were visually controlled. One of two CVD synthetic diamond-based target prototypes was irradiated for 30 min at the maximum current produced by the cyclotron in order to confirm the previously demonstrated performance during short irradiations.

### 3.3. Unique Vacuum System for Target Preparation

**Figure 3:**
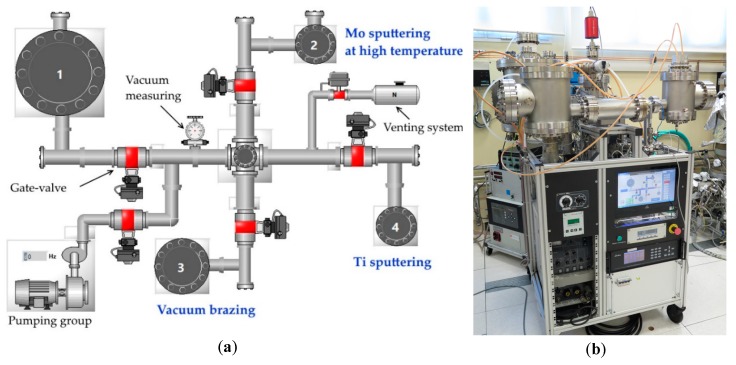
Vacuum system: (**a**) PLC control system layout and (**b**) photo of the complete system.

For target prototype preparation, a vacuum system consisting of four vacuum chambers connected through the central zone and separated by pneumatic gates was used in order to continue with both vacuum processes of interest in different chambers: sputter deposition and vacuum brazing (Figure 3). The central zone was connected to a general pumping system including a 360 L/min Pfeiffer turbo molecular pump with a Pfeiffer DCU display and operating unit (Pfeiffer Vacuum, Asslar, Germany), and a 210 L/min Varian (now Agilent, Santa Clara, CA, USA) Tri Scroll Pump as a primary pump. The base vacuum pressure of about 1 × 10^−6^ mbar was reached without additional backing before each experiment.

The entire system was controlled by a home-made human-machine interface LabVIEW (National Instruments Italy, Roma, Italy)-programmed PLC (Programmable Logic Controller). Figure 3a shows the four-chamber system layout used for PLC control. Chambers 2 and 4 were used for sputter deposition with 2″ magnetron sources. Chamber 3 was used for vacuum brazing.

The base vacuum pressure was controlled by a full-range Bayard-Alpert (BA) vacuum-meter. The pressure during MS deposition was controlled with a capacitance vacuum-meter since it was not sensitive to plasma as BA. The vacuum-meters were connected to a MaxiGauge^TM^ control box (Pfeiffer Vacuum, Asslar, Germany). The MKS (MKS Inc., Andover, MA, USA) multi-gas mass-flow controllers powered by the MKS 647C four-channel power supply/readout system was used for gas flow control during the deposition process. The entire system is shown in Figure 3b.

### 3.4. Magnetron Sputtering

**Figure 4:**
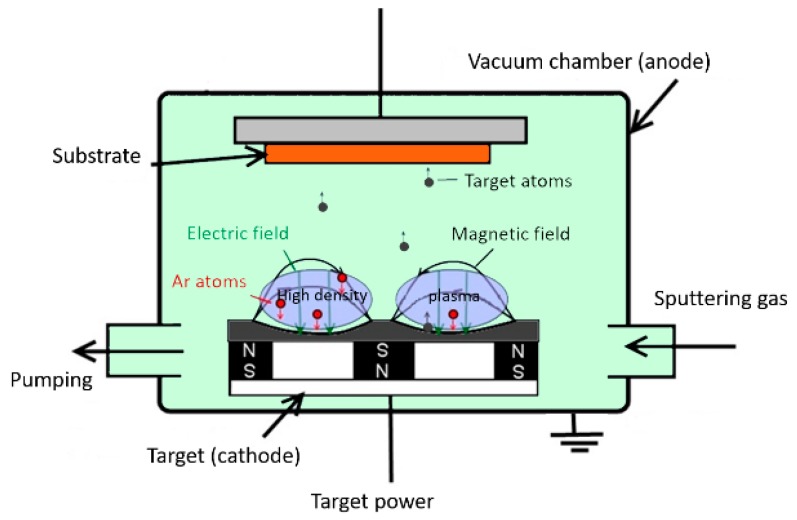
Schematic description of a magnetron sputtering process in down-top configuration.

Sputtering deposition occurs in a vacuum by means of inert gas plasma (Ar). The material to be deposited, called the sputtering target (not to be confused with the cyclotron target), is attached to the cathode. Plasma is created when a difference in potential is applied between the cathode and the substrate (anode). The positive ions of the inert gas are accelerated toward the cathode. When the ions collide with the atoms of the sputtering target, the energy transfer causes the detachment of some atoms, which are then deposited on the substrate. Magnetron sputtering is characterized by elevated plasma use efficiency due to its magnetic confinement. The process is schematically described in Figure 4.

In the current work, the films were deposited by direct current (DC) sputtering with a 2″ planar, unbalanced, II Type magnetron cathode source. The magnetron drive model MDX 1.5 kW (Advanced Energy, Fort Collins, CO, USA) was used to complete the sputtering experiments with the possibility of an automatic control in order to set up the pulsing mode to perform multilayer deposition.

Depositions were performed onto a planar substrate holder (SH), which had the ability to heat up the substrate by controlling the temperature with a K-type thermocouple inserted in the SH plate. The distance between the substrate and the cathode was 6 cm. For film deposition, we used the down-top deposition configuration with a magnetron source placed from the bottom of the cylindrical vacuum chamber and substrate holder with substrates from the top of the chamber in order to minimize film delamination caused by metallic dust particles.

Molybdenum was deposited on a spot 10 mm in diameter in the center of each substrate (backing plate) defined by an appropriate mask.

### 3.5. Thin Film Analysis

FEI (Philips, Amsterdam, The Netherlands) SEM XL–30 was used for sputtered coatings analysis.

The contact stylus profiler, model Dektak 8 (Veeco, Plainview, NY, USA), able to measure a surface texture below submicro-inch and film thickness up to 262 µm, was used for sputtered film thickness characterization.

### 3.6. Vacuum Brazing

The brazing process was performed in a small mobile homemade furnace placed inside a vacuum chamber. The standard vacuum ConFlat flange (nominal diameter 100 mm) with the assembled furnace and all electrical connections was placed downside in the vacuum chamber. The heating source in the furnace was a 450 W infrared (IR) lamp (Helios Italquarz, Cambiago-MI, Italy). The temperature was controlled by a K-type thermocouple placed inside the furnace with an automatic custom-made IR lamp backing control system. This system allowed us to control the heating temperature and the heating and cooling rate, since this aspect is extremely important for successful ceramics brazing. The small furnace reached the maximum temperature of 1050 °C.

In order to provide good thermal and mechanical contact during brazing, ceramic substrates were coated with 1–2 µm titanium deposited by DC magnetron sputtering at a 0.5-A DC current, 8.8 × 10^−3^ mbar Ar pressure at a 6 cm target-substrate distance in 20 min. The appropriate sputtering pressure was chosen in order to minimize the intrinsic stress in the film and avoid titanium film peeling.

Homemade paste was used as the filler material, containing 63% Ag, 35.3% Cu, and 1.7% Ti powder (content corresponding to CuSil active brazing alloy, ABA) mixed by three-dimensional shaker-mixer, model TURBULA^®^ T2F (Glen Mills Inc., Clifton, NJ, USA), water, and a water-soluble glue that was partially hydrolyzed under heating potato starch solution as a binder.

**Figure 5:**
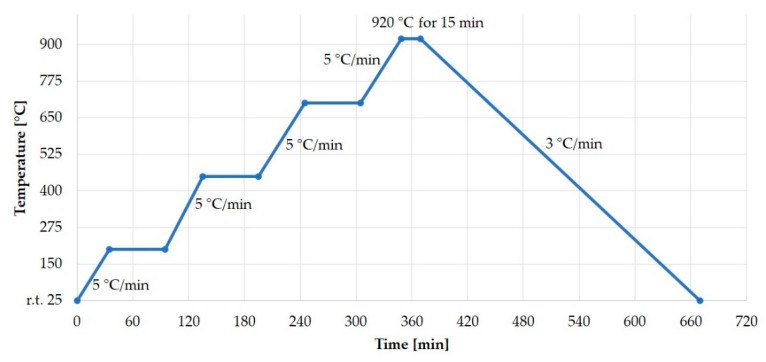
Thermal cycle for brazing sapphire and diamond plate to copper with CuSil ABA paste.

The brazing process was realized in a vacuum at 920 °C in 15 min maintaining 5 ° C/min heating and 3 °C/min cooling rate in order to minimize the thermomechanical stress in the final target assembly/system (Figure 5).

**Figure 6:**
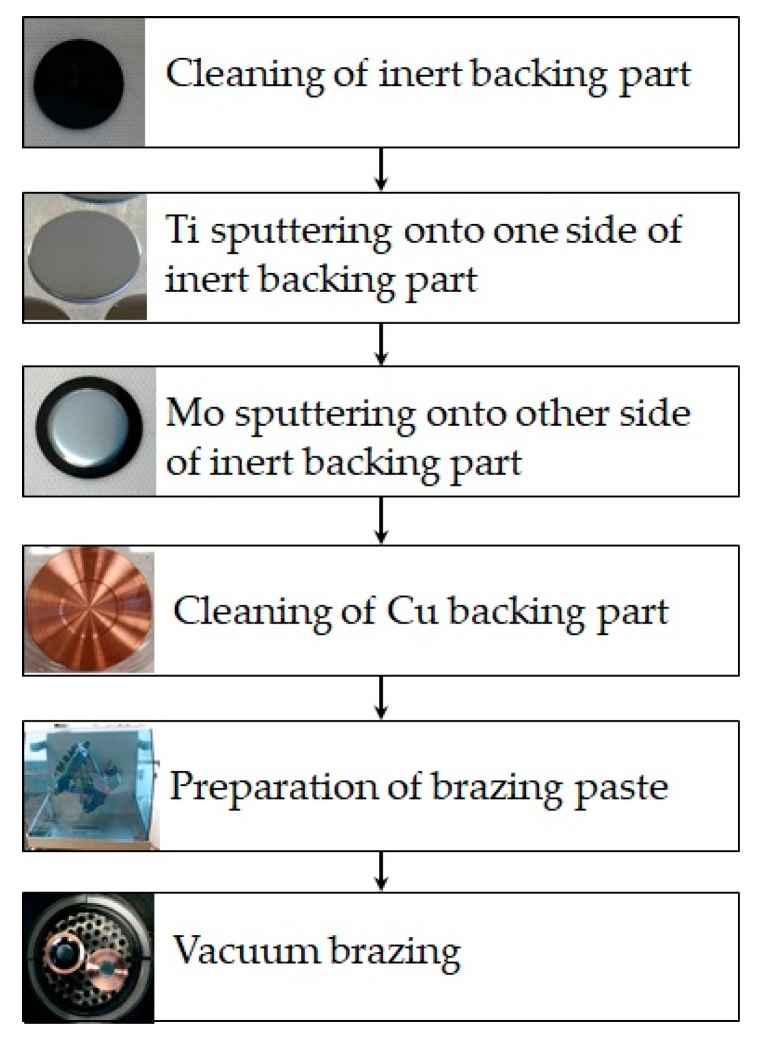
Innovative target prototype preparation steps.

The general process diagram used to realize the target prototypes is shown in Figure 6.

### 3.7. Dissolution Test and Gamma-Spectroscopy

In order to prove the chemical inertness of the ceramic materials used in the target prototype construction (sapphire and CVD synthetic diamond), the targets were exposed to standard dissolution conditions (concentrated hydrogen peroxide at 70 °C) [10] after cyclotron irradiation. The gamma spectroscopy analysis of an aliquot of the obtained solution was performed at the Research Laboratory of St. Orsola-Malpighi Hospital Medical Physic Department (Bologna).

The spectrometry system is based on a high purity Germanium detector with 30% relative efficiency and a resolution of 1.8 keV at 1332 keV. The samples were counted after a waiting time to allow for a decrease in radioactivity, sufficient to produce a total counting frequency <2000 counts per second in the 10–2000 keV energy range. The waiting time was typically within 1 h. The spectrometry system efficiency was calibrated in the 59–1836 keV range, using a multi radionuclide certified reference solution, obtained from an accredited Standardization Laboratory (LEA CERCA, Pierrelatte CEDEX, France). The calibration process was performed accordingly to the IEC 61452 standard, using Genie 2000 software (Version 3.2.1, Canberra Industries Inc., Canberra, Australia). A dual logarithmic polynomial efficiency curve was used. The propagation of uncertainties in the calibration considers the accuracy of the reference source (1–2% at 1 sigma level, depending on the peak in the mixture), the tabulated yield of peaks (typically <1%), the net peak area (<1% for calibration peaks), and the interpolation of the efficiency values. The latter is evaluated from the covariance matrix of the fitting of the efficiency curve (typically <3%). In total, using a quadratic propagation of the above terms, the calibration uncertainty was about 4–5% at the 1 sigma level.

## 4. Results and Discussion

### 4.1. Optimization of Magnetron Sputtering Parameters

The control of stress in physical vapor-deposited films is important due to its close relationship with material technological properties, adhesion strength to the substrate, and the limit of film thickness without cracking, buckling, or delamination [33]. In particular, for the purpose of this work, it was mandatory to avoid stressed films because poor adhesion between Mo films and the backing plate could drastically increase the thermal resistance of the contact, decreasing the heat exchange efficiency.

In this work, gas sputtering pressure and temperature of the holder were managed and optimized for the system configuration described above.

**Figure 7:**
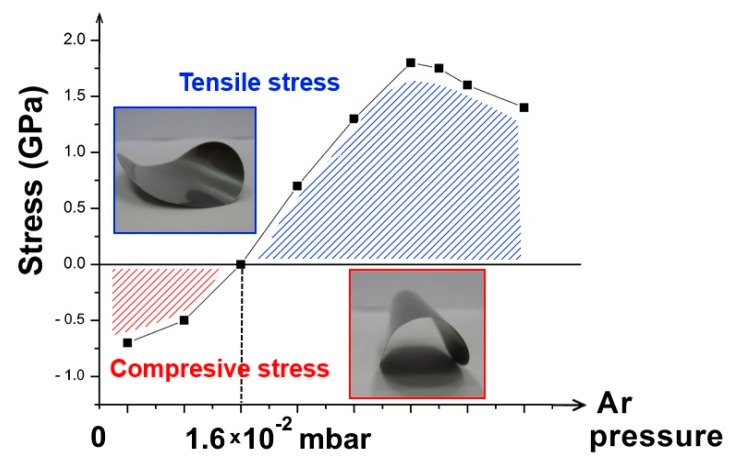
Stress vs. sputtering pressure.

Theoretically, a certain gas pressure corresponds to the transition between the tensile and compressive stress. At relatively high pressure, the frequency of the gas phase collision increases, reducing the kinetic energy of the sputtered atoms and reflected neutrals bombarding the growing film exhibiting an open porous microstructure; the interatomic attractive forces produce tensile stress. At low pressure, the arriving atoms have high kinetic energy and the resulting film has a dense microstructure, experiencing compressive stress [34]. The optimal pressure was obtained experimentally by performing depositions of Mo onto flexible substrates. The radius of curvature taken by the Kapton is an indicator of the stress (Figure 7).

**Figure 8:**
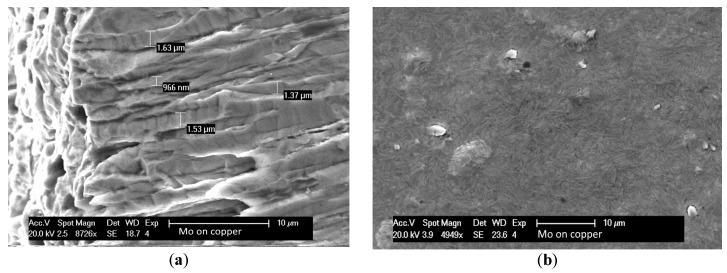
Scanning electron microscopy (SEM) analysis of (**a**) a cross-section and (**b**) top view of 90 µm Mo sputtered at 500 °C on copper.

The substrate temperature influences the kinetic energy of the particles that have already arrived at the substrate. Low temperature promotes the columnar voided microstructure that is associated with tensile stress. High temperature corresponds to an increase in adatom mobility that leads to a bulk-like structure [35]. In this work, we performed the deposition at the homologous temperature T_h_ = T/T_m_ = 0.2, where T is the temperature during vacuum deposition and T_m_ is melting point of material deposited. The high temperature of the holder during the Mo sputtering process provided a microstructure with a density of more than 95% of the bulk material (Figure 8). The Mo film had a columnar microstructure with a grain size from several hundred nm to microns as confirmed by the SEM image in Figure 8a.

A multilayer deposition technique was shown to reduce stress [36], thus the deposition of Mo thick films was fragmented in a thousand consecutive brief depositions of thin films using an automation program to control the power. Each deposition was followed by a “relaxation time” during which the film was annealed (80% duty cycle for a one-minute period).

The best sputtering process parameters to obtain unstressed Mo films onto copper, sapphire, and CVD diamond are listed in Table 2. ^nat^Mo films of about 98% of bulk density with thickness over 100 µm were successfully deposited onto copper, sapphire, and synthetic diamond, which were further used either as ready target prototypes (Cu-1,2,3 and S-1,2) or for further vacuum brazing to create target prototypes (S-3,4,5 and D-1,2).

### 4.2. Irradiation

**Figure 9:**
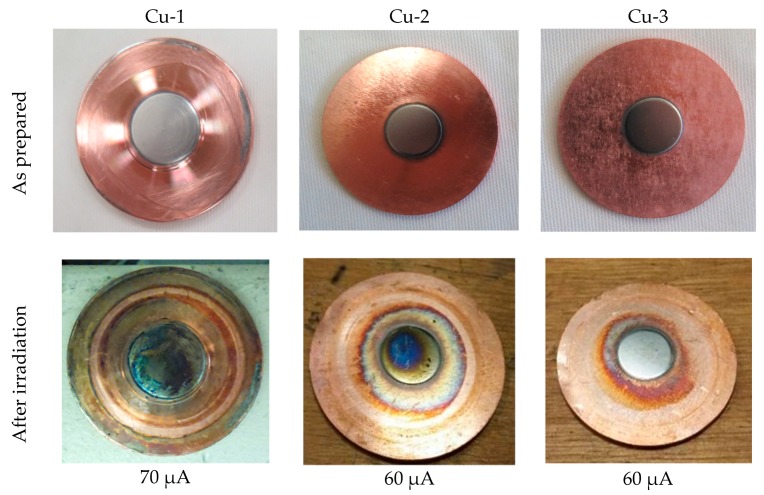
Mo on copper backing target prototype irradiation test.

**Figure 10:**
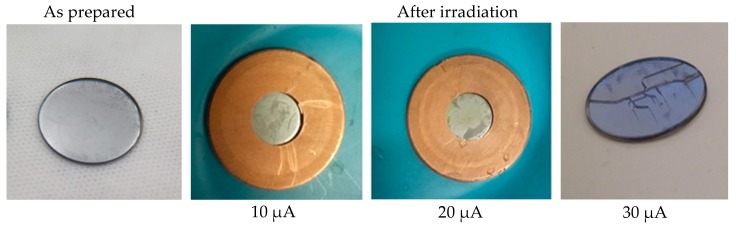
Irradiation test of S01 Mo sputtered on sapphire target prototype inside a four-pin target holder.

**Figure 11:**
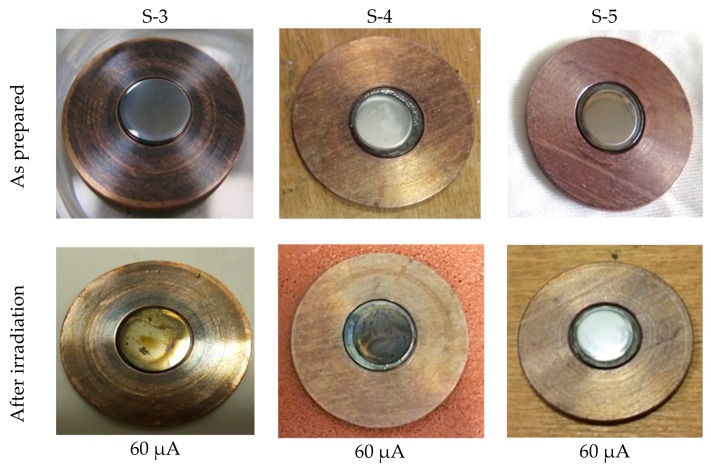
Irradiation test of Mo sputtered on a sapphire-based complex backing prototype.

After irradiation, the targets were visually inspected, looking for any sign of melting, checking the overall integrity of the target, and the adherence of the Mo film to the backing materials. The results of the irradiation are summarized in Table 3 and visual details are shown in Figure 8, Figure 9, Figure 10 and Figure 11.

The 110–125 µm of Mo sputtered directly onto copper backing targets were irradiated for 1 min at 15.6 MeV energy on the target (after the Havar^®^ foils) and currents 30, 50, 60, and 70 µA. No sign of target damage was observed after irradiation at 30 and 50 µA. The oxidation of the Mo and Cu surfaces (observed in Cu-1 and 2) was related to the previous leakage of a small amount of cooling water in the target system. The copper target test proved that sputtered Mo film resists at a 70 μA irradiation current, maintaining excellent contact between the Mo film and Cu backing (Figure 9).

We place 90 µm of natural Mo on a sapphire coin (sample S–1 and S–2) inside the four-pin target holder for irradiation at 10, 20, 30, and 50 µA for 1 min. The Mo film on sapphire resisted a beam current of 10 µA and 20 µA and the sapphire remained intact. At 30 µA current, sapphire-backed plate cracked but the Mo film kept the pieces together, without evidence of delamination or oxidation (Figure 10). At 50 µA, the sapphire cracked into two pieces (one of them was used for the dissolution test described below). It is clear that the contact between sapphire and the copper four-pin holder was extremely poor. A better contact is essential in order to perform the production at higher proton beam currents.

The prototype S-3, composed of 110 µm Mo sputtered onto 12.7-mm-diameter and 0.5-mm-thick sapphire, brazed onto copper, was tested in several steps by increasing the current in each following run, from 30 µA to the maximum cyclotron current of 60 µA, always at maximum beam energy 15.6 MeV. Each irradiation test lasted for 1 min. No cracking of the sapphire piece occurred. The prototypes S-4 and S-5 were tested directly under the maximum current of 60 µA at 15.6 MeV for 1 min. The systems resisted the proton beam irradiation as shown in Figure 11. Notably, in the time between the prototype S-4 preparation and irradiation test, a defect between the sapphire and brazing filler occurred, which seems to be attributed to a problem with the Ti sputtered film adherence before the brazing process of the sapphire. For the following preparation of diamond-based prototypes, the Ti sputtering parameters were optimized and additional attention was paid to substrate cleaning procedures in order to avoid this problem.

**Figure 12:**
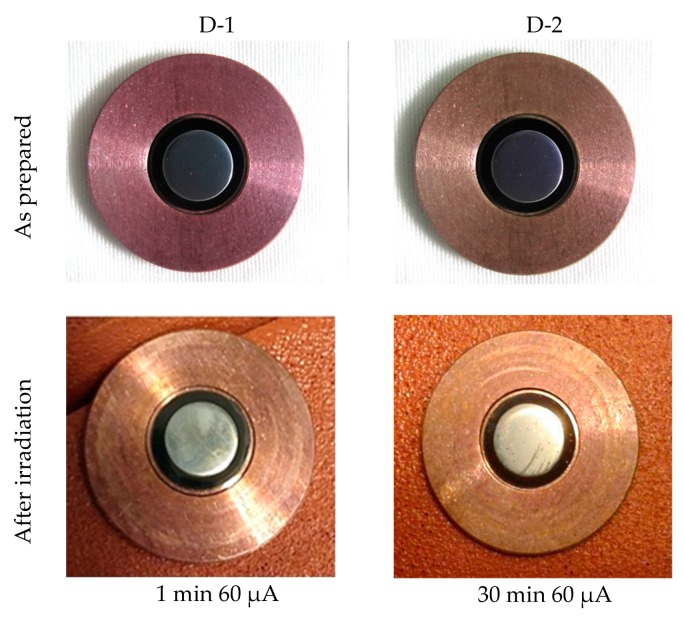
Irradiation test of Mo sputtered on a synthetic diamond–based complex backing prototype.

The first CVD synthetic diamond-based prototype D-1 was tested in several steps by increasing the current in each following run from 20 µA to 40 µA, and finally at maximum cyclotron current of 60 µA. A maximum entrance beam energy 15.6 MeV was used. Each irradiation test lasted 1 min. No cracking or oxidation of the target prototype occurred during irradiation as shown in Figure 12.

One irradiation test on the D-2 CVD synthetic diamond-based prototype was performed under conditions closer to those used in actual production. The irradiation was performed for 30 min at 60 µA and 15.6 MeV. Similar to the first diamond-based prototype, the second one showed excellent performance during the long irradiation test without any signs of damage (Figure 12).

The new cyclotron target prototype composed of ^nat^Mo sputtered on synthetic diamond brazed to Cu backing produced excellent results from a thermomechanical point of view, remaining stable at heat power densities in the order of 1 kW/cm^2^.

It should be noted that the cyclotron current typically used for electrodeposited solid targets (for example, ^64^Ni on gold backing for ^64^Cu production [37,38,39] and ^68^Zn on platinum backing for ^68^Ga production [40]) ranges from 20 to 50 µA, corresponding to a maximum heat power density of about 0.5 kW/cm^2^. Hence, the use of the sputtering process for target preparation can increase the radiopharmaceutical production yield two-fold. This demonstrates the better performance of the sputtered targets in respect to those produced by electrodeposition.

### 4.3. Dissolution Test and Chemical Inertness Prove

The test of the chemical inertness of the ceramic part of the backing involved dissolution followed by γ-spectroscopy analysis.

A piece of S-2 irradiated target was dissolved and 10 µL of solution were placed in the γ-spectrometer in order to control if any impurities were released by the irradiated sapphire. The range of the γ-ray energy was 80–4096 keV. According to the software, the peaks belonging to ^92m^Nb, ^94^Tc, ^95^Tc, ^95m^Tc, ^96^Tc, ^99^Mo, and ^99m^Tc were identified (Table 4). All radionuclides are the irradiation products of natural molybdenum. ^92m^Nb is produced by the reaction ^95^Mo(p,α); all other radionuclides are produced by a set of (p,xn) reactions starting from the natural molybdenum isotopes: ^92^Mo, ^94^Mo, ^95^Mo, ^96^Mo, ^97^Mo, ^98^Mo, and ^100^Mo [8,41]. Therefore, any contaminant radionuclides produced by the sapphire backing were present in the solution. This proves the chemical inertness of sapphire in the chosen dissolution conditions.

The thermal grade CVD diamond (D-0 sample), Ø 13.5 mm and 0.3 mm thick with thermal conductivity 1500 W/(m·K), was tested under the 15.6 MeV and 20 µA cyclotron accelerated proton beam for 1 min inside the four-pin target-holder. After irradiation, the synthetic diamond piece was placed into concentrated H_2_O_2_. No change in the mass with respect to the mass of the diamond before dissolution was observed. The piece of synthetic diamond was then used for the γ-spectroscopy analysis to identify the elements created inside the diamond piece under irradiation. The γ-spectrum contained only one peak at 511 keV. The 511 keV is the energy of the photons due to the electron-positron annihilation. Thus, the radioactive isotope produced in the synthetic diamond during the proton irradiation was a positron emitter. The only suitable radioisotope is ^13^N decaying to ^13^C, emitting a positron. In order to prove this, the activity from the sample was measured via dose-calibrator versus time to build a decay curve. The time corresponding to the moment when 50% of activity left was 10 min. This means that T_1/2_ = 10 min. The radioisotope produced in CVD synthetic diamond was ^13^N.

No other products besides ^13^N were detected in the CVD synthetic diamond plate after 15.6 MeV proton beam irradiation. The synthetic diamond plate does not release any foreign radionuclides after irradiation according to γ-spectroscopy analysis.

### 4.4. Magnetron Sputtering Efficiency and Further Perspectives

The main defect of the magnetron sputtering technique is the low efficiency of the deposition, which means high losses of enriched material. The losses are attributed to two main factors: low sputtering target use in standard configuration and losses of the sputtering chamber. The first can be solved using an advanced technique with High Target Utilization plasma Sputtering (HiTUS) [42]. The method for ultra-thick film sputtering proposed here can be easily applied to different sputtering techniques since it is based on main PVD principles. For the other factor, an efficient method to recover enriched Mo material from the sputtering chamber after deposition is required. The LARAMED group of LNL-INFN has already developed a method for enriched Mo recovery based on dissolution in an ammonium hydroxide and peroxide, precipitation of MoO_3_, and further molybdenum oxide reduction in an overpressure hydrogen reactor.

Positive results obtained for ^nat^Mo targets have created potential for the application of the magnetron sputtering technique for a high current target with non-enriched elements as precursors. As an example, the LARAMED group of LNL-INFN has successfully developed a preparation method for ^nat^Y target for the production of ^89^Zr.

## 5. Conclusions

In this work, we developed a sputtering deposition process of a target material, producing Mo films more than 100 µm thick with bulk-grade density, low oxidation level, and high adherence to the backing plate, in order to create solid targets for medical radioisotope production. Due to the versatility of the magnetron sputtering technique, target prototypes were created by direct deposition on both metallic (copper) and non-metallic (sapphire and CVD synthetic diamond) target backing.

The use of sapphire and CVD synthetic diamond as backing materials guarantees chemical inertness during the dissolution process after irradiation in order to minimize the impurities. In order to reduce the implementation costs, a composite backing plate made of a non-metallic part vacuum brazed to copper was suggested. In order to produce such prototypes, the vacuum brazing method of sapphire and CVD synthetic diamond to copper was successfully developed using home-made brazing paste.

Solid cyclotron target prototypes, both on simple copper backing and on complex backing based on sapphire or CVD synthetic diamond substrates, showed excellent thermomechanical stability under 15.6 MeV and 60 µA (maximum available from used PETtrace cyclotron) proton beam, corresponding to a heat power density of about 1 kW/cm^2^.

## 6. Patents

From the work reported in this manuscript, an Italian patent application No. 102017000102990, dep. ref. P1183IT00, inventors V. Palmieri, H. Skliarova, S. Cisternino, M. Marengo, G. Cicoria, title “Metodo per l’ottenimento di un target solido per la produzione di radiofarmaci”, was applied by Istituto Nazionale di Fisica Nucleare on 14.09.17; and extended to the International patent application PCT/IB2018/056826, dep. ref. P1183PC00, on 07.09.18, title “Method for obtaining a solid target for radiopharmaceuticals production”.

## Figures and Tables

**Table 1:** State-of-the-art cyclotron targets for production of ^99m^Tc (preparation and irradiation).

Country	Institution	Cyclotron Type	Target-holder System	Irradiation Conditions	Target Material	Backing Material	Deposition Process	Problems Notes	Ref.
Saudi Arabia	KFSHand RC ^1^	CS-30, 15–23 MeV	Cooled by both water and He, channels, beam at 90° to target.	1 µA 10 min in 2013; 15 µA already burnt	^nat^Mo, oxide, ^100^Mo	Cu, Al	Hydraulically pressing ^nat^Mo, ^100^Mo oxides powder into the circular cavity target plates, heating (450–400 °C)	Only the low current test was satisfactory. ^nat^Mo burns.	[4,5]
Canada	University of Alberta	TR24 ACSI ^2^ 500 µA, 24 MeV p+	-	-	Mo metallic	Ta, W, glassy carbon, quartz, Al_2_O_3_	1600 °C sintering in reducing atmosphere, 600 °C press bonding to a backing	Not tested	[5]
		TR19 ACSI, 19 MeV p+	Rectangular inclined	100 µA	Mo metallic	Al_2_O_3_	1600 °C sintering in reducing atmosphere, 600 °C press bonding to a backing	No information about tests	[4]
		TR24 ACSI 500 µA, 24 MeV	TR24 standard, rectangular inclined	150–500 µA	Mo metallic	Al	Foils composed of powders rolled, annealed, hot pressed to backing	The backing is not inert as claimed	[4]
		TR19 ACSI, 19 MeV p+	Rectangular inclined	20–30 µA	Mo metallic	Al	Hydraulically pressed to backing	The backing is not inert as claimed	[14]
		TR19/9 ASCI, 19 MeV p+	30 ° to beam	15.5/18 MeV up to 95 µA max 71 µA average on target 360 min	Mo metallic	Al, Ag, Pt, Au, Ta, Ti, V, Ni, Zn, Zr, Nb, Ru, Rh, Pd, Ir	Foils, pressing to a backing, melting on Ta backing, 1600 °C sintering in reducing atmosphere, 400–500 °C press bonding to the backing of Ta, Cu, preliminary oxidized Al in H_2_O_2_ and HNO_3_	The backing is not inert as claimed. No clear data on Al pre-oxidized.	[14,15,16]
	TRIUMF	TR19 ACSI	Patented ACSI TR19 target station: both inclined to beam with microchannels and perpendicular to the beam	80–300 µA 16–30 MeV 0.5–8 h; 10.8 kW, 0.6 kW/cm^2^, 300 µA 18 MeV beam spot 10 × 20 mm 10° to beam. (< 500 °C on a plate)	Mo metallic oxidation-reduction-Mo metallic refined	Ta, other transition metals are described but not claimed	Electrophoretic deposition from a mixture of refined Mo powders 10 µm + molybdate + binder onto Ta backing, sintering 1200–1900 °C 38 h in an inert atmosphere (final receipt 1700 °C 5 h)	85% density; Ta backing is not inert as claimed	[4,5,6,25]
		TR24/TR30 ACSI	TR30 target	24 MeV. 500 µA, 2.1 kW, 1.2 kW/cm^2^	Mo metallic	Ta		Ta backing is not inert as claimed	[4]
		GE PETtrace	GE PETtrace	16.5 MeV 130 µA, 5.6 kW, 0.3 kW/cm^2^	^100^Mo metallic powders	GLIDCOP ^3^ 30 mm D 1.3 mm thick, brazed to 20 mm 0.7 mm thick Mo	Mo sintered at 1300–2100 °C disc 18.5–19.5 mm diameter 0.6 mm thick, placed in GLIDCOP baseplate and brazed in a vacuum, H_2_/Ar at 500–1000 °C by Ag-Cu-P	No chemical inertness	[4,9,17]
				-	-	-	Electrochemical deposition up to 20 µm Mo from aqueous solution 10M acetate	Deposition inefficient <2%	[21]
	ACSI	TR19 ACSI	Inclined	15–52 µA 1.5–3 h 15.5–17 MeV	^100^Mo powders	Ta	Direct melting onto a backing	No data on thickness and performance. Nonuniform thickness.	[19]
	Isopor-Isotopos Para Diagnostico E Terapeutica S.A.	Low energy cyclotron	-	-	^100^Mo metallic or oxide		3-layer sandwich: substrate 40–1500 µm/^100^Mo layer 25–500 µm/2nd hard core layer(Al, Ag, Cu) 25–500 µm/1st hard core layer (Nb, Pt, Ta, Ag, Havar)	Strange and unclear target concept	[30]
Japan	Fukui Medical University, Matsuoka	-	Vertical beam	-	-	Target vessel for in situ preparation (Alumina)	-	The production of ^89^Zr by ^89^Y(p,n) was investigated; not tested for Tc	[5]
	Japan Atomic Energy Agency, Ibaraki	Nuclear reactor	-	(n,γ) reaction	MoO_3_ pellet	Irradiation container	Plasma sintering method + oxidation process	Low thermal conductivity material	[11]
	MIC NIRS ^4^	-	Horizontal beam	-	3–5 mm layered ^100^MoO_3_	Target vessel for irradiation, dissolution	Preparation of 3–5 mm thick ^100^Mo oxide layer: ^100^Mo + H_2_O_2_ + N_2_ flow in a target vessel	Enriched powder; lower ^99m^Tc yield/higher contaminants	[4]
		-	Vertical beam	-	Powder ^100^Mo as purchased, without solidification	SiC target vessel	-	Limited information	[4]
Armenia		Cyclotron C18	Nitra commercial solid target system	-	Mo/MoO_3_ powder pellet + Ag powder compound	-	-	Limited information	[5,12]
	A. I Alikhanyan National Science Laboratory	Cyclotron C18	Target is fixed by pneumatic clamps; (work in progress--> cryogenic cooling to use 100 µA)	18 MeV; proton current 30 µA	Tablet of ^−nat^Mo	Solid state target disk: Nb or Ti	Compression method and tablet surface burning method using a focused laser beam to increase mechanical strength and additional treatment to provide adhesion with the backing	No irradiation test	[4]
India	VECC ^5^	Low power cyclotron	Circular target	8–18 MeV, 10–50 nA, 5 min	^nat^Mo foils 25 µm	Cu spacers	A stack of 4–7 25 µm foils with Cu foil monitor	Very low currents	[4,5]
				1–6 h, 1–3 µA, 8–18 MeV	^nat^Mo powders	-	980 MPa pressed powders	Very low currents	[4,5]
Italy	JRC-Ispra ^6^	Scanditronix MC40 K = 38	Al cylindrical screw-type target station devoted to stack foil irradiation	100 nA, 1 h, 8–21 MeV	^100^Mo powders in foils	Al degraders, target holder of Al	Stacked foils technique alternating Al and enriched Mo and Ti foils. Mo foils prepared by electron-beam melting in nitrogen, slow cooling, lamination with pack-rolling technique	Low currents	[5,18]
	LNL-INFN ^7^	GE PETtrace	The first prototype of TEMA solid target	70 µA, 16 MeV, 30 min, 1 kW/cm^2^	^nat^Mo bulk	Cu	Magnetron sputtering of ^nat^Mo	For the moment work only with ^nat^Mo	[5]
Iran	RRDL, NSTRI ^8^	Cyclone-30	Inclined target	160 µA, 25 MeV, 1000 µA-h	^nat^Mo	Cu	Thermal spray 130 µm Mo	Not inert backing	[26]
Poland	RC POLATOM ^9^	GE PETtrace 870	2.5/5.7 µA for ^100^Mo target	-	^nat^Mo	Pt	Electrodeposition from aqueous solutions	Only oxide is deposited	[5]
					^nat^Mo powders	-	Pressing, sintering pellet, preliminary H_2_ treatment 166 °C; 50–78% density After H_2_ treatment was improved	Considerable oxide-low thermal conductivity	[4,5]
		-				-	Electrodeposition from molten salts LICl-NaCl-KCl-MoCl_3_ 600 °C on Ni plate in argon gave low oxidation	No data on thickness and quality	[4]
		-					Mechanical reshaping: melting, remelting, rolling, annealing in vacuum. Different thickness 0.25–600 µm. H_2_ treatment before remelting. Heating in an envelope of SS.	Lamination technological difficulties	[31]
	Institute of Metallurgy and Mat. Sci.				Mo-Zn alloy		Mo-Zn alloys co-electrodeposited from citrate solutions	Not pure Mo	[22]
USA		–	–	–	–	Pt	Electroplating from alkaline solutions	A mixture of oxide and metal. Not pure metal	[20]
	Washington University	CS–15	–	10–15 MeV 3,4,5µA	^100^Mo_2_C	Pt	^100^Mo_2_C synthesized from ^100^MoO_3_ using 3 steps thermal carburization method; pressed on Pt target holder (5000 psi for 30 s)	Low beam current and irradiation time	[4,5]
	Massachusetts Institute of Technology	–	–	–	–	–	Electrodeposition: Pre-electrolysis + Electrodeposition of Mo. K_3_MoCl_6_ (source of soluble Mo) and KCl (a principal constituent of the supporting electrolyte) –> thickness 0.5 mm, columnar structure, but the presence of protrusion on the surface	No limitation on the thickness, but the process is complicated and contaminants are present.	[23]
Syria	–	–	Inclined elliptical IBA blank copper target	–	MoO_2_	Cu	Electroplating from aqueous solution.	A very thin layer, poor adherence, oxidation	[4,5]
				–	natMoO3 oxide	Cu	Pressing, sintering 750 °C 50 µm MoO_3_	Backing not inert	[5]
				100 µA, 3 h	^nat^Mo powders	Cu	Pressing Mo powders	Backing not inert	[4]
Germany	GSI ^10^	–	–	–	^100^Mo, ^92^Mo, ^98^Mo	C or Cu	FIB sputtering with Ar+ Sletten–Type apparatus. 0.1/0.14 µm films also self–sustaining	Small thickness reported	[27]
	Sektion Physik Universitat Munchen	–	–	–	^100^Mo, ^98^Mo	Cu backing or self–sustaining	High vacuum sputter deposition with 10 keV Xe + gun. 100–400 nm thick films/foils	100–400-nm-thick films/foils	[29]
UK	Mallinckrodt Llc ^11^	–	–	–	^100^Mo metallic, MoO_3_ or their combination	–	Target obtained from commercial supplier (probably material)	No data on irradiation	[13]
	Daresbury Laboratory	–	–	–	^100^Mo	–	Rolling from powders. Thickness is about 0.5 and 10 µm thick	About 0.5 and 10 µm thick	[28]
Romania	Institute of physical chemistry	–	–	–	0.01 mm quite dense, adherent.	Metal	Electrodeposition from molten salts NaCl-KCl-NaF K_2_MoO_4_ 1123 K on Ni plate in argon produced low oxidation	Difficult preparation, expensive equipment	[24]

^1^ KFSH and RC—King Faisal Specialist Hospital and Research Center, Saudi Arabia;

^2^ ACSI—Advanced Cyclotrons Systems Inc. (Richmond, Canada);

^3^ GLIDCOP (Höganäs AB, Sweden);

^4^ MIC NIRS—Molecular Imaging Center of National Institutes of Radiological Science, Chiba, Japan;

^5^ VECC Variable Energy Cyclotron Centre, Kolkata, India;

^6^ JRC-Ispra—Joint Research Centre, Ispra, Italy;

^7^ LNL-INFN—Legnaro National Laboratories, Italian National Institute for Nuclear Physics, Legnaro, Italy;

^8^ RRDL, NSTRI—Research and Development Lab, Nuclear Science and Technology Research Institute, Tehran, Iran;

^9^ RC POLATOM—Radioisotopic Centre POLATOM, Otwock, Poland;

^10^ GSI—GSI Helmholtz Centre for Heavy Ion Research, Darmstadt, Germany;

^11^ Mallinkrodt Llc, Staines-upon-Thames, United Kingdom.

**Table 2:** Optimized sputtering process parameters.

Sputtering Parameter	^nat^Mo
Argon flux (sccm)	17
Argon pressure (mbar)	1.63 × 10^−2^
Power (W)	5–550
Target-substrate distance (cm)	6
Substrate temperature (°C)	500
Deposition rate (µm/h)	11
Program for multilayer	Yes

**Table 3:** Irradiation test (energy 15.6 MeV).

Prototype Name	Ceramic (Inert) Part	Ceramic Dimensions (mm)	Mo Film Thickness (µm)	Irradiation Current (µA)	Irradiation Time (min)	Notes
Cu-1	–	–	110	30	1	Resisted
				50	1	Resisted
				70	1	Resisted
Cu-2	–	–	125	60	1	Resisted
Cu-3	–	–	125	60	1	Resisted
*S-1	Sapphire	Ø12.7 × 0.5	90	10	1	Resisted
				20	1	Resisted
				30	1	Cracked
*S-2	Sapphire	Ø12.7 × 0.5	90	50	1	Cracked
S-3	Sapphire brazed	Ø12.7 × 0.5	110	30	1	Resisted
				40	1	Resisted
				60	1	Resisted
S-4	Sapphire brazed	Ø12.7 × 0.5	125	60	1	Cracked
S-5	Sapphire brazed	Ø12.7 × 0.5	125	60	1	Resisted
* D-0	Diamond	Ø13.5 × 0.3	–	20	1	Resisted
D-1	Diamond brazed	Ø13.5 × 0.3	125	20	1	Resisted
				40	1	Resisted
				60	1	Resisted
D-2	Diamond brazed	Ø13.5 × 0.3	125	60	30	Resisted

* Inside 4-pin target holder.

**Table 4:** Gamma spectrum analysis of proton-irradiated Mo sputtered on sapphire.

Nuclide Name	ID Confidence	Energy (keV)	Yield (%)
Nb–92m	0.998	934.46	99.00
Tc–94	0.902	702.62	99.60
		849.74	95.70
		871.09	100.00
		1592.10	2.25
Tc–95	0.992	765.79	93.82
		947.67	1.95
		1073.71	3.74
Tc–95m	0.999	204.12	63.25
		582.08	29.96
		820.62	4.71
		835.15	26.63
Tc–96	0.985	314.34	2.43
		778.22	100.00
		812.58	82.00
		849.93	98.00
		1091.35	1.10
		1126.96	15.20
Mo–99	0.981	140.51	89.43
		181.06	5.99
		739.50	12.13
		777.92	4.26
Tc–99m	0.936	140.51	89.00
